# A Close Look at Hearing Repair, One Protein at a Time

**DOI:** 10.1371/journal.pbio.1001584

**Published:** 2013-06-11

**Authors:** Richard Robinson

**Affiliations:** Freelance Science Writer, Sherborn, Massachusetts, United States of America

Hearing is a matter of transducing mechanical forces into electrical signals. Pressure waves coming to the ear drum are transmitted through the middle ear to the mechanosensory hair cells within the fluid-filled inner ear. The “hairs” (more properly called stereocilia) on a hair cell are arranged in semicircular rows, with the shortest at the front and the tallest at the back. An incoming wave deflects the stereocilia backward, causing protein chains called tip links, which link the tip of a short stereocilium to the side of a taller one, to pull on and open up ion channels at the base of the protein chains. The influx of ions from the surrounding fluid causes hair cell depolarization and firing of the attached sensory neurons.

**Figure:**
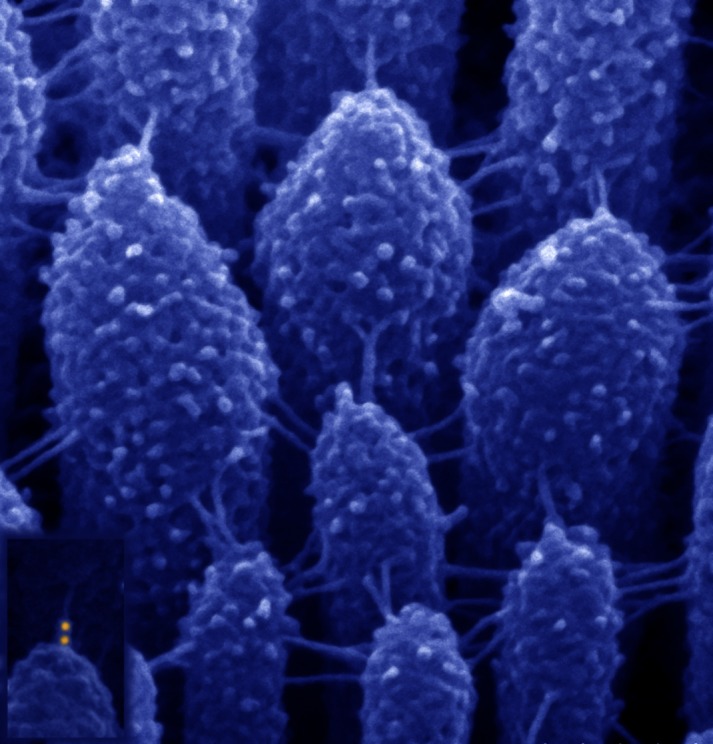
The inner ear transduces sound via tip links that open up ion channels; loud sounds can break tip links, but their regeneration is possible through a two-step remodeling process of their molecular components. Image credit: Gregory I. Frolenkov.

Exposure to excessively loud noises—a jet engine, a rock concert—leads to temporary hearing loss. One cause of that loss is breakage of those tip links, which is not surprising, given both their central importance and relative fragility. Tip links re-form over the course of several days, restoring normal hearing. While the molecular composition of tip links is known, the details of regeneration have remained unclear. In a new study in *PLOS Biology*, Artur Indzhykulian, Gregory Frolenkov, and colleagues begin to reveal those details, showing that of the two components of the mature link, one is solely responsible for the initial re-formation of the chain, and that full restoration of normal hearing only occurs once the second protein is back in place.

The two proteins are known as protocadherin 15 (PCDH15), which on the mature tip link is found closest to the shorter stereocilium, and cadherin 23 (CDH23), which links to the longer stereocilium (mutations of the genes for both these proteins cause inherited deafness). The authors treated mouse hair cells with calcium-free solution to disrupt all the links connecting the stereocilia, and then observed tip link regeneration, using scanning electron microscopy. Over the first several hours, new links formed only at the top of the stereocilium, rather than forming at the base and migrating, as an early hypothesis of regeneration had suggested.

Mechanotransduction, measured by deflecting stereocilia with a rigid probe and measuring the properties of the resulting current, was abolished by the calcium-free solution. Current flow gradually returned over the next 24 hours, accompanied by restoration of the full complement of tip links. But a property of the mechanotransduction response called fast adaptation (in which activation is followed by rapid deactivation) remained aberrant, before gradually normalizing beginning at about 36 hours.

This normalization appeared to be connected to changes in the molecular composition of the restored tip links. The authors found that immediately after disruption, PCDH15 was redistributed along the surface of the stereocilia. In contrast, CDH23 largely disappeared from them. As the links were reformed, the total amount of PCDH15 in the tip link area increased, while the amount of CDH23 remained relatively low compared to untreated controls. Furthermore, PCDH15 was found at both ends of the links, not just the lower end, as in controls. This suggested that PCDH15 was serving, at least temporarily, in place of CDH23 as links reformed. Then, after the links were in place, CDH23 was gradually restored, coincident with recovery of normal adaptation.

Some physiologists have speculated that the repair process recapitulates the developmental formation of tip links. In support of that idea, the authors found that in normal development in postnatal in mice, PCDH15 was localized at both ends of the tip link at postnatal day 1, and 5 days later, it was concentrated mainly at the lower end. Over the same period, CDH23 remained only at the upper end.

One outcome of this study is to resolve a small dispute over the composition of tip links, in which one camp argued for a PCDH15-only structure, and the other for a combination of the two proteins. Apparently, both are right, depending on when one looks. More importantly, it reveals the details of a process vital for the development, maintenance, and restoration of normal hearing.

**Indzhykulian AA, Stepanyan R, Nelina A, Spinelli KJ, Ahmed ZM, et al. (2013) Molecular Remodeling of Tip Links Underlies Mechanosensory Regeneration in Auditory Hair Cells. doi:10.1371/journal.pbio.1001583**

